# X-Linked Lissencephaly With Absent Corpus Callosum and Abnormal Genitalia: An Evolving Multisystem Syndrome With Severe Congenital Intestinal Diarrhea Disease

**DOI:** 10.1177/2329048X17738625

**Published:** 2017-11-07

**Authors:** David Coman, Tom Fullston, Cheryl Shoubridge, Richard Leventer, Flora Wong, Simon Nazaretian, Ian Simpson, Josef Gecz, George McGillivray

**Affiliations:** 1 Victorian Clinical Genetics Services, Murdoch Children’s Research Institute, Royal Children’s Hospital, Melbourne, Victoria, Australia; 2 School of Medicine, The University of Queensland, Brisbane, Queensland, Australia; 3 School of Medicine, Griffith University, Gold Coast, Queensland, Australia; 4 Department of Genetic Medicine, Women’s and Children’s Hospital, North Adelaide, South Australia, Australia; 5 School of Paediatrics and Reproductive Health, University of Adelaide, Adelaide, South Australia, Australia; 6 Department of Neurology, Royal Children’s Hospital, Melbourne, Victoria, Australia; 7 Murdoch Children’s Research Institute, Melbourne, Victoria, Australia; 8 Department of Pediatrics, University of Melbourne, Melbourne, Victoria, Australia; 9 Department of Newborn Services, Monash Medical Centre, Melbourne, Victoria, Australia; 10 Department of Anatomical Pathology, Monash Medical Centre, Melbourne, Victoria, Australia

**Keywords:** XLAG, lissencephaly, agenesis of the corpus callosum, diarrhea, pancreatic insufficiency, enteroendocrine cells, *ARX* gene, congenital intestinal diarrheal diseases

## Abstract

X-linked lissencephaly with abnormal genitalia is a rare and devastating syndrome. The authors present an infant with a multisystem phenotype where the intestinal manifestations were as life limiting as the central nervous system features. Severe chronic diarrhea resulted in failure to thrive, dehydration, electrolyte derangements, long-term hospitalization, and prompted transition to palliative care. Other multisystem manifestations included megacolon, colitis, pancreatic insufficiency hypothalamic dysfunction, hypothyroidism, and hypophosphatasia. A novel aristaless-related homeobox gene mutation, c.1136G>T/p.R379L, was identified. This case contributes to the clinical, histological, and molecular understanding of the multisystem nature of this disorder, especially the role of *ARX* in the development of the enteroendocrine system.

X-linked lissencephaly with abnormal genitalia (MIM 300215) was first described by Berry-Kravis and Israel.^1^ More than 20 affected males have subsequently been reported.^1–12^ Consistent clinical features (see Table 1) include the following: (1) a brain malformation characterized by lissencephaly and agenesis of the corpus callosum, (2) perinatal encephalopathy with an intractable seizure disorder, (3) hypothalamic dysfunction manifest by poor temperature regulation, (4) ambiguous or underdeveloped genitalia, and (5) death in early infancy, usually within 3 months of age.^4^ Common neuroanatomical features of X-linked lissencephaly with abnormal genitalia include the following: (1) mildly increased cortical thickness of approximately 6 to 10 mm, (2) distinctive histopathology reflecting defective tangential migration of precursor γ-aminobutyric acid (GABA)-producing interneurons, (3) a posterior–anterior gradient of severity, (4) complete agenesis of the corpus callosum with or without associated interhemispheric cyst, (5) basal ganglia dysplasia, (6) enlarged posterior temporal horns of the lateral ventricles (colpocephaly), and (7) normal brain stem and cerebellar development.^4-5,13–16^

**Table 1.** Clinical Manifestations in XLAG.^a^

Commonly Reported in XLAG	Feature	References
Morphological	High forehead	^2,4^
	Large anterior fontanelle	^2,4^
	Mildly low set ears	^2,4^
	Ambiguous or underdeveloped genitalia	^2,4^
Neurological	Lissencephaly with a posterior>anterior severity gradient	^4^
	Complete absence of the corpus callosum	^4^
	Dysplastic basal ganglia	^4^
	Intractable seizure disorder	^4^
	Hypothalamic dysfunction	^2,4^
Gastrointestinal	Chronic diarrhea	^7,9,10,13,14^, present case
Less Commonly Reported in XLAG	Feature	References
Cardiorespiratory	Mild left lung hypoplasia	^3^
	Ventricular septal defect	^3^
	Patent ductus arteriosus	^3,6,9^
	Patent foramen ovale	^2,6^
Endocrine	Hypothyroidism	^6,7,9^, present case
Gastrointestinal	Nonspecific active colitis	Present case
	Mild megacolon	^3,9^, present case
	Exocrine pancreatic insufficiency	^7,9^
Biochemical	Hypophosphatemia	^7,9^, present case

Abbreviation: XLAG, X-linked lissencephaly with abnormal genitalia.

^a^ “Common in XLAG” = reported in ≥50% of reported patients, “less common in XLAG” = reported in <50% of reported patients.

De novo and inherited *ARX* mutations have been demonstrated in males with X-linked lissencephaly with abnormal genitalia and in some of their female relatives.^5^ The Aristaless-related homeobox (*ARX*) gene (MIM 300382) is located at Xp22.13. The *ARX* encodes a 562 amino acid protein that localizes to the nucleus and functions as a transcription factor.^5,13^ The ARX is expressed in the developing hypothalamus, thalamus, basal ganglion, and cerebral cortex where it plays a key role in promoting the tangential migration of interneurones expressing GABA.^5,13^ Similar transcription regulatory activities in the endocrine pancreas and tests are required for normal pancreatic and Leydig cell development, respectively (see Figure 1). The X-linked lissencephaly with abnormal genitalia is a multisystem disorder, with nonneurological clinical features including hypothyroidism, chronic diarrhea, megacolon, exocrine pancreatic insufficiency, and hypophosphatasia (see Table 1). The neurological manifestations of X-linked lissencephaly with abnormal genitalia are undoubtedly devastating; the authors report a case where the gastrointestinal manifestation of chronic diarrhea was life limiting and resembled that seen in the congenital intestinal diarrheal diseases. The complication of congenital intestinal diarrheal diseases in our patient resulted in the need for ongoing inpatient care for life-threatening fluid loss and nutritional compromise. It ultimately led to the parent’s request for palliative care because of their concerns about their son’s quality of life.

**Figure 1.**
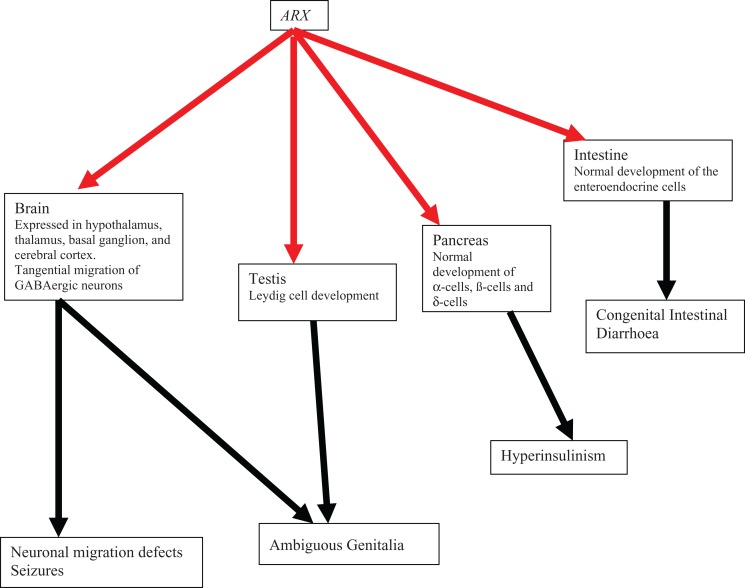
The ARX roles in embryogenesis and phenotypes. Solid red arrows indicate roles in normal embryogenesis. Solid black arrows depict phenotypes consequent to perturbations in *ARX* function in embryogenesis. ARX indicates aristaless-related homeobox.

## Case Report

The proband was the first child to nonconsanguineous parents. An 18-week gestation obstetric ultrasound scan was reported normal. The 36-week scan suggested severe ventriculomegaly, prompting delivery by Cesarean section at 37 weeks’ gestation. The newborn infant’s growth parameters were between the 10 and 50th percentiles for gestation (birth weight: 2745 g, height: 48.5 cm, head circumference: 33.5 cm). He required bag-and-mask ventilation with Apgar scores of 5 (1 minute) and 8 (5 minutes).

Clinical examination of a newborn revealed marked generalized hypotonia with brisk reflexes, mildly dysmorphic features with a high forehead, a large anterior fontanelle and mildly low set ears, ambiguous genitalia with a micropenis (length: 10 mm, diameter: 8 mm), a fused scrotum, and cryptorchidism. Repeat neurological examination at age 6 weeks showed a head circumference of 34 cm (<1st percentile). The baby was extremely irritable and would not fix or follow. There was axial hypotonia with marked head lag and appendicular hypertonia with brisk limb reflexes and sustained clonus at both ankles. Frequent generalized myoclonic jerks were noted. A severe, refractory seizure disorder, resistant to multiple anticonvulsants (phenobarbitone, phenytoin, topiramate, sodium valproate, clobazam, and clonazepam) was evident on the first day of life. Electroencephalography showed continuous bilateral, multifocal epileptic discharges and a paucity of normal background rhythms, at times approaching a burst suppression pattern.

**Figure 2.**
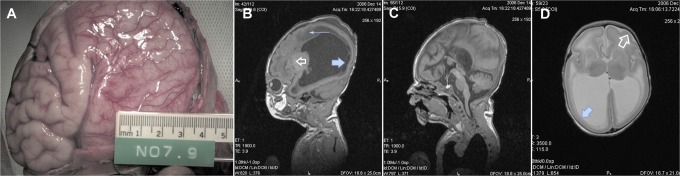
Brain pathology and MRI findings in XLAG: (A) cerebrum at autopsy reveals posterior lissencephaly with transition to anterior pachygyria, (B) sagittal T1 MR sequence demonstrates lissencephaly P>A and marked lateral ventriculomegaly. Note subependymal cysts (large arrow) abnormal periventricular white matter signal (small arrow) and abnormal basal ganglia signal clear arrow, (C) sagittal T1 MR sequence of the midline demonstrates agenesis of the corpus callosum, (D) axial T2 MR sequence demonstrates colpocephaly and lissencephaly with transition from posterior agyria (solid arrow) to anterior pachygyria (open arrow). MR indicates magnetic resonance; XLAG, X-linked lissencephaly with abnormal genitalia.

Magnetic resonance imaging (MRI; Figure 2) identified lissencephaly with agyria posteriorly transitioning in the mid-parietal region to pachygyria anteriorly, with a maximal cortical thickness of 10 mm in the occipital lobes, agenesis of the corpus callosum, and marked dilation of the bodies, atria, and temporal horns of the lateral ventricles. Microcysts were identified bilaterally in the putamen of the basal ganglia. A nonspecific parenchymal cyst was identified adjacent to the right anterior temporal horn. The white matter, thalami, brain stem, and cerebellum appeared normal.

Hypothalamic dysfunction was demonstrated by poor temperature regulation and hypothyroidism (free thyroxine 14.1 pmol/L [range: 25-70 pmol/L]; thyroid-stimulating hormone 1.37 IU/L [range: 1-25 IU/L]). Thyroxine replacement therapy was commenced. Mild elevations in liver enzyme and bilirubin levels were noted, with an elevated alkaline phosphatase 930 IU/L (100-350 IU/L), γ-glutamyl transferase 1201 IU/L (0-40 IU/L), serum bilirubin 30 IU/L (0-10 μmol/L), and conjugated bilirubin 14 μmol/L (0-5 μmol/L). These changes persisted and were thought related to hypothyroidism. Hypophosphatemia with a serum phosphate of 0.92 mmol/L (1.3-2.3 mmol/L) was noted in the first week of life. Banded karyotype was normal 46, XY.ish17p13.3 (LISIx2), as were skeletal radiographs, renal ultrasound scans, and an ophthalmologist’s examination. Echocardiography revealed a small patent foramen ovale with patent ductus arteriosus.

Chronic diarrhea was a major management issue, resulting in failure to thrive, dehydration, metabolic acidosis, and hypokalemia. The patient spent 7 out of the first 8 weeks of life in hospital consequent to the chronic diarrhea. There was presumptive evidence of small bowel malabsorption secondary to exocrine pancreatic insufficiency. An initial stool sample tested positive for fat globules with weakly positive tryptic activity. Alfare C24 formula was commenced at 3 weeks of age with improved stool consistency and some weight gain. The patient was discharged at a month of age for supported home care. He was admitted 2 days later with dehydration requiring intravenous fluids due to ongoing severe diarrhea. Fat globules were again positive. Fecal tryptic activity was negative. Fecal-reducing substances were not detected. He was discharged at 6 weeks of age weighing 3029 g for supported home care on Alfare C24 and 1% Liquigen. The diarrhea worsened with time resulting in hypernatremic dehydration (sodium 168 mmol/L) with a mild metabolic acidosis. Faced with persisting diarrhea and refractory seizures, the parents requested a palliative care approach from 2 months of age. The patient died at 3 months of age, consequent to the severe diarrhea and fluid loss.

Consent for a targeted autopsy was granted. Macroscopic examination of the brain confirmed pachygyria of the frontal lobes with abrupt transition to agyria of the parietal, temporal, and occipital lobes with the cortex demonstrating an intermediate thickness of 8 to 10 mm. Histology revealed a generalized reduction in the number of cortical neurons in the frontal, temporal, parietal, and occipital lobes and in the basal ganglia, due to neuronal dropout and apoptosis. Some cortical organization was present, but cortical layering was ill defined. Sections of the brain stem revealed heterotopic neurons within the pyramidal tracts and other parts of the brain stem. The white matter was hypercellular with gliosis, and heterotopic neurons were again observed. Agenesis of the corpus callosum was confirmed. The cerebellum was normally formed, with histological features indicative of terminal ischemia. Additional histopathological studies of brain tissue were considered retrospectively but were not able to be performed.

Pancreatic sections showed nesidioblastosis confirmed with Neoplastic spindle cell (NSC) staining. There was no histological evidence of exocrine pancreatic insufficiency. Hepatic architecture was normal, with small bile plugs indicating bland canalicular cholestasis. Histology of the small bowel was normal. Sections of colon indicated a patchy neutrophil infiltrate, occasional cryptitis, and crypt abscess formation consistent with a nonspecific colitis. Mild megacolon was evident at the rectosigmoid junction with an external diameter of 15 mm. Normally formed ganglion cells were present in the submucosal and myenteric plexi.

An apparently de novo and novel single-base substitution, c.1136G>T, was identified in exon 4 of *ARX*, resulting in the substitution of an arginine to leucine amino acid substitution at position 379 (p.R379L). The mutation was not identified in DNA extracted from maternal blood and the mother’s MRI brain scan showed a normally formed corpus callosum and brain.

## Discussion

The *Arx* is a homeobox gene which is highly expressed in the forebrain, floor plate, and testis of the mouse X-linked lissencephaly with abnormal genitalia model,^5^ and mutations in this gene exhibit a pleiotropic range of clinical phenotypes: XL West syndrome, XL myoclonic epilepsy with spasticity and intellectual disability, Partington syndrome, and nonsyndromic intellectual impairment.^17^ The X-linked lissencephaly with abnormal genitalia phenotype is generally associated with mutations/alterations leading to a premature truncation of the ARX protein or affecting highly conserved residues in the homeodomain.^13^ The authors identified a novel *ARX* mutation, c.1136G>T (p.R379L), in exon 4. The arginine residue at position 379 in ARX is conserved across paired-type homeodomain proteins and is part of a nuclear localization sequence.^18,19^ The authors anticipate that the c.1136G>T mutation in *ARX* leading to the p.R379L substitution can disrupt binding of ARX to importin 13 and subsequently affect the efficient localization of ARX protein to the site of action in the nucleus or correct distribution once inside the nucleus. Alternatively, this p.R379L substitution can affect the binding of ARX to DNA. Either mechanism would be predicted to disrupt important transcriptional regulatory events during GABAergic interneuron proliferation, differentiation, and migration and contribute to the severe neurological phenotype observed in this patient.

While the clinical phenotype of X-linked lissencephaly with abnormal genitalia is dominated by the severe neurological malformations, our case highlights the importance of considering X-linked lissencephaly with abnormal genitalia as a truly multisystem disease process (Table 1). Chronic diarrhea has previously been reported in at least half of the reported patients with X-linked lissencephaly with abnormal genitalia.^7,9-10,14^ The congenital intestinal diarrheal diseases group of diseases are classified into 4 broad etiological groups based on the underlying pathophysiology; (1) defects of digestion, absorption, and transport of nutrients and electrolytes; (2) defects of absorptive enterocyte differentiation or polarization; (3) defects of the enteroendocrine cells; and (4) defects of the immune system affecting the intestine.^20^

The intestinal enteroendocrine cells have a key role in the regulation of food intake, glucose homeostasis, digestion, and absorption.^20,21^ Defects of the enteroendocrine system are causative for the congenital intestinal diarrheal diseases in a small number of Mendelian diseases, congenital malabsorptive diarrhea (MIM 610370), prohormone convertase 1/3 deficiency (MIM 600955), and autoimmune polyendocrinopathy–candidiasis–ectodermal dystrophy syndrome (MIM 240300). There is emerging evidence that *ARX* is essential for normal enteroendocrine cell development.

Approximately half of the patients with X-linked lissencephaly with abnormal genitalia and missense or nonsense mutations in *ARX* present with congenital diarrhea.^14,22^ Insight into the pathogenesis of the congenital intestinal diarrheal diseases phenotype is reproduced by the mouse *Arx* model which also demonstrates diarrhea and failure to thrive secondary to a loss of enteroendocrine cell subpopulations.^21,23^ ARX/Arx is required for the specification of a subset of enteroendocrine cells in both humans and mice, with loss of ARX/Arx leading to altered enteroendocrine differentiation, most notably reductions in cholecystokinin, glucagon-like peptide 1, and somatostatin populations.^21,22^ Mild megacolon was evident at the rectosigmoid junction in our patient, and the presence of ganglion cells is consistent with reports in 2 other patients.^3,9^

The *ARX* is a transcriptional initiator and repressor and plays key role in normal pancreatic endocrine cell development.^24^ The *Arx* is known to be expressed in the mouse pancreas and islets of Langerhans cells.^25^ Mutations in murine *Arx* lead to a histological decrease in pancreatic α-cells and increases in β cells and δ cells.^25,26^ Deficiencies of the glucagon- and pancreatic polypeptide-producing cells in the islet of Langerhans have been demonstrated in patients with X-linked lissencephaly with abnormal genitalia,^26^ providing further evidence to the importance of *ARX* in the normal human pancreatic development.^24^ Nesidioblastosis was identified on pancreatic histology samples from our patient, which to date has not been reported in patients with X-linked lissencephaly with abnormal genitalia. The persistence of fecal fat globules and the subsequent absence of tryptic activity in our patient are presumptive evidence that small bowel malabsorption secondary to exocrine pancreatic insufficiency was an important etiological factor. Exocrine pancreatic insufficiency has been described in 2 previous cases^7,9,26^; however, the diarrhea did not abate with either pancreatic enzyme replacement or total parenteral nutrition with bowel rest.^7^ Similar findings have been observed in a 2-year-old with X-linked lissencephaly with abnormal genitalia.^10^ In this case, vasoactive intestinal peptide was normal, and the administration of octreotide was effective in relieving the diarrhea. Hypothalamic dysfunction is common in X-linked lissencephaly with abnormal genitalia; the authors are only aware of one other X-linked lissencephaly with abnormal genitalia case with documented hypothyroidism.^7^ Adrenal gland hypoplasia, however, has been identified at autopsy.^4^ Mutations in *Arx* block Leydig cell differentiation, leading to severely dysgenetic testicles,^3^ which do not respond in a functionally appropriate manner to hormone stimulation.^11^ This, rather than hypothalamic dysfunction, is thought to account for the genital phenotype.

The X-linked lissencephaly with abnormal genitalia is a devastating clinical disease whose phenotype is dominated by severe neurological manifestations; however, our case highlights that the X-linked lissencephaly with abnormal genitalia clinical phenotype is truly a multisystem disorder where the nonneurological manifestations can be as severe as the neurological features. The authors conclude that X-linked lissencephaly with abnormal genitalia should be included in the Mendelian diseases known to be associated with congenital intestinal diarrheal diseases from enteroendocrine dysgenesis.
